# Reactivity of HLADR antibody manifests expression of surface MHC II molecules on peripheral blood T lymphocytes in new world monkeys

**DOI:** 10.1002/iid3.1318

**Published:** 2024-06-24

**Authors:** Sriram Chitta, Bharti P. Nehete, Ashley B. Delise, Joe H. Simmons, Pramod N. Nehete

**Affiliations:** ^1^ Department of Comparative Medicine, Keeling Center for Comparative Medicine and Research UT‐MD Anderson Cancer Center Bastrop Texas USA; ^2^ Department of Comparative Medicine The University of Texas Graduate School of Biomedical Sciences Houston Texas USA

**Keywords:** flow cytometry, MHC Class II, New World Monkeys, T lymphocytes, whole blood

## Abstract

**Background:**

Major histocompatibility complex (MHC) class II molecules expressed on B cells, monocytes and dendritic cells present processed peptides to CD4^+^ T cells as one of the mechanisms to combat infection and inflammation.

**Aim:**

To study MHC II expression in a variety of nonhuman primate species, including New World (NWM) squirrel monkeys (*Saimiri boliviensis boliviensis*), owl monkeys (*Aotus nancymae*), common marmosets (*Callithrix spp*.), and Old World (OWM) rhesus (*Macaca mulatta*), baboons (*Papio anubis*).

**Methods:**

Two clones of cross‐reactive mouse anti‐human HLADR monoclonal antibodies (mAb) binding were analyzed by flow cytometry to evaluate MHC II expression on NHP immune cells, including T lymphocytes in whole blood (WB) and peripheral blood mononuclear cells (PBMC).

**Results:**

MHC class II antibody reactivity is seen with CD20^+^ B cells, CD14^+^ monocytes and CD3^+^ T lymphocytes. Specific reactivity with both clones was demonstrated in T lymphocytes: this reactivity was not inhibited by purified CD16 antibody but was completely inhibited when pre‐blocked with purified unconjugated MHC II antibody. Freshly prepared PBMC also showed reactivity with T lymphocytes without any stimulation. Interestingly, peripheral blood from rhesus macaques and olive baboons (OWM) showed no such T lymphocyte associated MHCII antibody reactivity.

**Discussion & Conclusion:**

Our results from antibody (MHC II) reactivity clearly show the potential existence of constitutively expressed (with no stimulation) MHC II molecules on T lymphocytes in new world monkeys. These results suggest that additional study is warranted to evaluate the functional and evolutionary significance of these finding and to better understand MHC II expression on T lymphocytes in new world monkeys.

## Graphical abstract

MHC II expression on T cells from New world monkeys, not on rhesus and baboon

## 1 INTRODUCTION

Major histocompatibility complex (MHC) class II proteins are expressed on the surface of professional antigen presenting cells (APC) like dendritic cells (DCs) and B cells. When MHC class II molecules are loaded with peptides derived from invading pathogens, they migrate from intracellular compartments to the cell surface to interact with CD4 T cells for activation.^1^, ^2^ Peripheral blood monocytes express low levels of MHC II proteins and upregulate MHC II molecules that are stably loaded with peptides when differentiated into DC.^3^, ^4^, ^5^, ^6^, ^7^, ^8^ MHC class II proteins are occasionally associated with CLIP (Class II‐associated invariant chain peptide)^9^, ^10^ or remain empty, peptide free,^11^, ^12^, ^13^ when expressed on the surface of immature DC or nonprofessional APCs.

T lymphocytes interact with peptides present on APCs and are activated to elicit a T cell response and CD4 T cells recognize peptides presented by MHC II protein. Expression of MHC II molecules on nonprofessional APC, including T lymphocytes, is inducible and interferon gamma (IFNγ) is often used for induction of MHC II expression.^14^ In humans MHC class II molecules are expressed on activated T cells,^15^ CD8 cytotoxic T cells (CTL) in HIV patients,^16^ effector CD4 T cells in active tuberculosis patients,^17^ and on regulatory T cells.^18^ Though MHC II expression is reported in T cell lymphoma, its expression is defective in leukemic and other T cell malignancies due to epigenetic control of CIITA (class II, MHC, transactivator).^19^ In animals also, expression of MHC II molecules on T cells is shown in rat,^20^ canine,^21^ equine,^22^, ^23^ porcine,^24^ and bovine.^25^ In equine, increased expression of MHC class II on T cells has been suggested as a marker of the memory phenotype.^23^ Mice are exceptionally distinct in the absence of MHC II expression on T cells even by activation. The reconstitution of MHC class II expression in mouse T cells with B cells fusion and complementary DNA transfection experiments showed a defective CIITA is responsible for absence of MHC II expression on mouse T cells.^26^

The heterodimer MHC II proteins are encoded by A and B genes and MHC polymorphism is confined to the B gene; however, both alpha and beta subunits of MHC II proteins contribute to the peptide binding grove.^27^ The serological, biochemical and immunoprecipitation methods used in classification of human MHC II subtypes were also employed in characterization of MHC class II in nonhuman primates using cross‐reactive human antibodies.^28^, ^29^, ^30^, ^31^, ^32^, ^33^ The molecular information including haplotype analysis, sequence homology and evolutionary significance of MHC II in NHP species documented in literature^34^, ^35^, ^36^ and as found in humans, β‐chain is variable (polymorphic) in NHP species.^31^, ^33^ Phenotyping nonhuman primate immune cells using cross‐reactive human monoclonal antibodies by flow cytometry, has helped to better understand nonhuman primate models used in biomedical research.^37^ The cross reactivity of human antibodies varies across nonhuman primate species and differs between Old‐World Monkey (OWM) and New World Monkey (NWM).^37^, ^38^, ^39^, ^40^, ^41^, ^42^ Cross‐reactive human antibody reactivity helps to monitor changes in antigen expression and absolute number of immune cells when nonhuman primates are used in biomedical research.^43^, ^44^ MHC protein expression in NHPs, as in humans, is important in antigen presentation to T cells, particularly when NHPs are used as models to understand immune responses in pathogenic infections and to evaluate the efficacy of human vaccines.^45^, ^46^ Here we used two clones of human MHC II antibody to analyze binding to immune cells in whole blood (WB) and fresh peripheral blood mononuclear cells (PBMCs) by flow cytometry. We showed a definitive expression of MHC II molecules on T lymphocytes, in addition to B cells and monocytes in squirrel, owl, and marmoset monkeys, but not on rhesus macaques or olive baboons. Though limited in evaluating the nature and function of MHC II expression on T lymphocytes, our results are first in nature to show constitutive expression of MHC II molecules on surface of T lymphocytes in NWM (squirrel, owl, and marmoset).

## 2 MATERIALS AND METHODS

### 2.1 Whole blood

Ethylenediaminetetraacetic acid treated peripheral blood samples were obtained from animals housed at the Keeling Center. Animals (squirrel monkey [SQM], owl monkey [OM], and marmoset) were not sedated to draw the blood samples. The blood samples for all animals (including rhesus and baboon) were obtained from samples that were otherwise to be discarded after clinical laboratory analyzes.

### 2.2 Antibody staining

WB was stained according to a previously described protocol.^41^ Briefly, conjugated mouse antihuman antibodies for antigen markers CD3 (clone SP34), CD20 (clone L27), and CD14 (clone M5E2) were added to 100 µL of blood in a 12 × 75 mm polystyrene tube. Two clones of conjugated MHC II antibodies, L243 (BioLegend) and LB3.1 (gift from Dr Lawrence J Stern, UMASS Medical School, Worcester, MA) were used as per the experiments. After incubation in the dark at room temperature (RT) for 20 min, red blood cells were lysed using 1× BD RBC lysis buffer. Cells were then washed with FACS buffer (1× phosphate‐buffered saline [PBS] containing fetal bovine serum [FBS] and sodium azide) and fixed in paraformaldehyde (1%) buffer for acquisition.

### 2.3 Flow cytometry

Antibody‐stained cells were acquired using a BD FACS Celesta (BD Biosciences) with FACS Diva software. Single color compensation controls were prepared with beads (ebiosciences) for each flow test used. Isotype controls were used wherever appropriate and when antibodies/clones were used for the first time. Approximately 50,000 events covering both lymphocytes and monocyte gated population were collected. FlowJo software (BD biosciences) was used for analysis of MHC II antibody binding on CD3^+^ T cells and CD20^+^ B cells from lymphocyte population and CD14^+^ monocytes from the monocyte population.

### 2.4 Blocking and binding specificity of MHC II antibody reactivity with T lymphocytes

A total of 100 µL of WB incubated with 10 µg of purified antibodies for IgG2a (isotype), CD16 (IgG FcgRIIIa), and MHC II antibody for 20 min at RT. After preincubation, the antibody staining of WB with conjugated antibodies was performed using fluorochrome conjugated antibodies for CD3 and MHC II for flow cytometry analysis. Results are shown as flow cytometry quadrant plots and graphs.

### 2.5 PBMC isolation

PBMC were isolated from fresh WB using a Ficoll gradient protocol. WB was diluted two‐fold with PBS (Ca^2+^Mg^2+^ free pH7.4) and layered over Ficoll. The sample was centrifuged at 500*g* for 30 min at 20°C with no break. The leukocyte layer was collected as PBMC and was washed a couple times with PBS. Cells obtained after the final wash were counted and adjusted to 1 × 10^7^/mL in PBS and used for staining PBMC were stained following the protocol described for WB, except that PBMC were incubated with antibody on ice for 30 min and cells were washed couple of times directly with cold FACS buffer. The rest of the steps for flow cytometry and analysis were the same as used for WB samples.

### 2.6 Data analysis and presentation

Data figures were created using FlowJo (v10.8.0) and GraphPad Prism (v 9.0.0). One‐way analysis of variance used to obtain *p* values (GraphPad Prism software v 9.0.0).

## 3 RESULTS

### 3.1 Human MHC II antibody reactivity with peripheral blood immune cells in SQM

**Figure 1:**
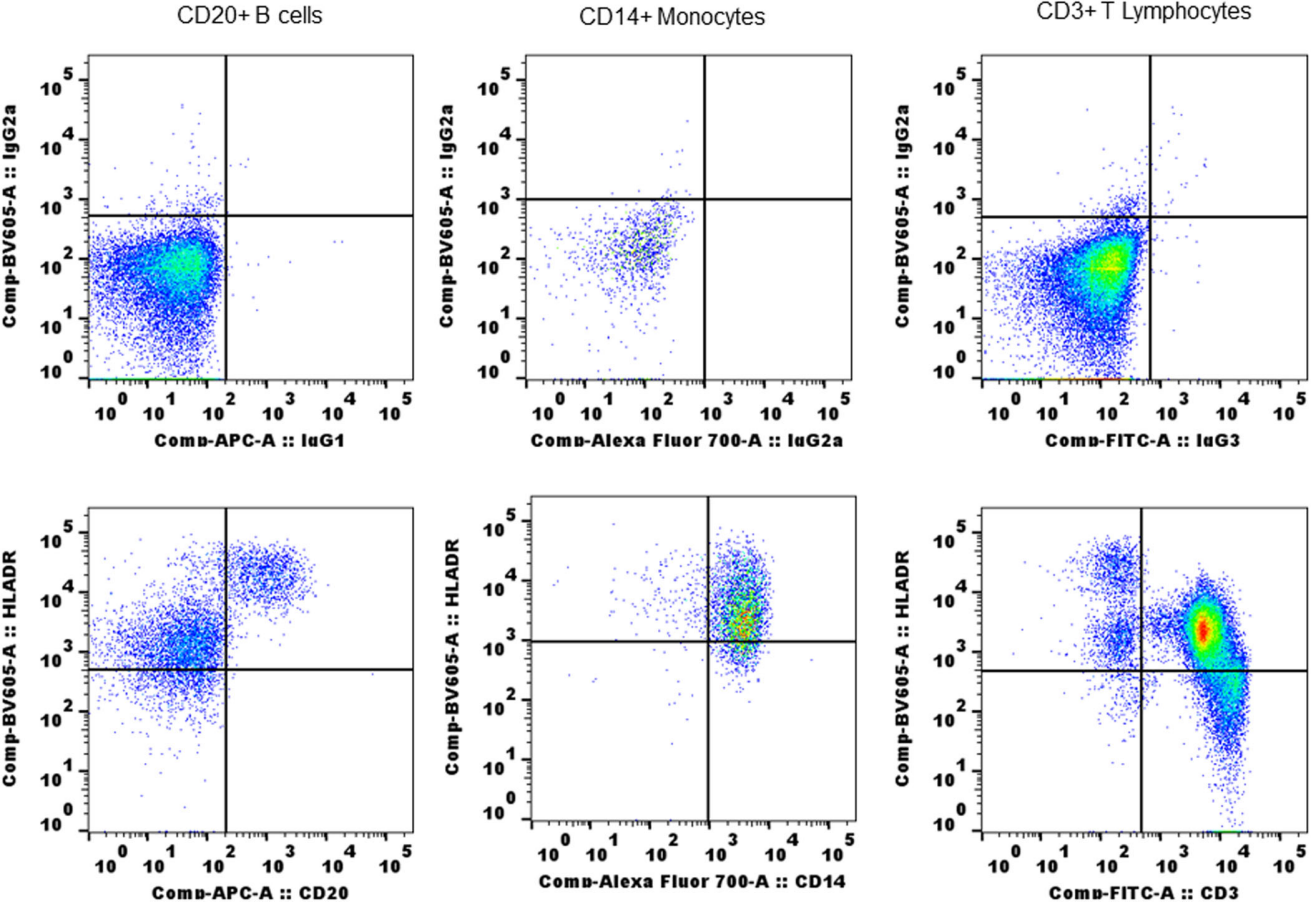
Reactivity of human MHC II antibody (clone L243) with peripheral blood immune cells: 100 µL of EDTA whole blood stained with cocktail of CD20, CD14, and CD3 antibodies along with MHC II antibody. Upper panel shows whole blood stained with isotype controls. Staining protocol and flow cytometry including analysis were described in Section 2. EDTA, ethylenediaminetetraacetic acid; MHC, major histocompatibility complex.

**Figure 2:**
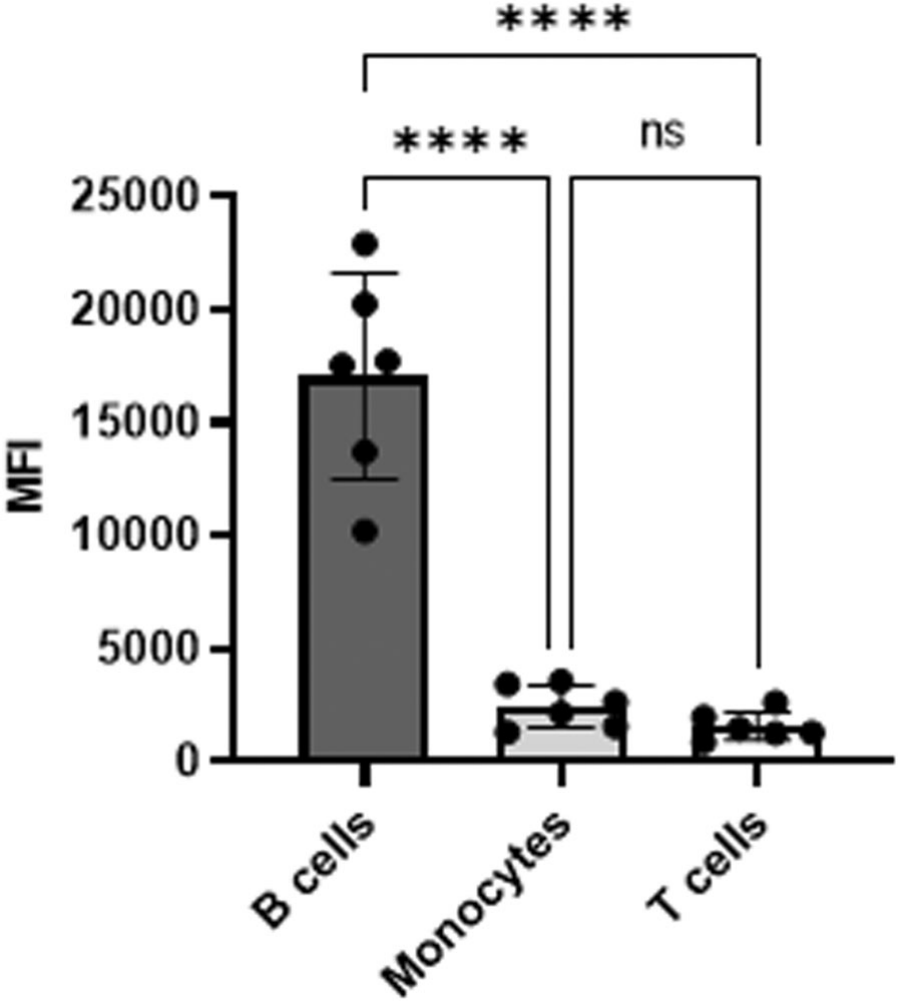
Mean fluorescence intensity (MFI) of cross reactive antihuman HLADR antibody (clone L243) staining with T cells, B cells and monocytes in squirrel monkey: CD3^+^ T cells, CD20^+^ B cells and CD14^+^ monocytes were identified from lymphocytes and monocytes gates from FSC/SSC plot, using specific surface markers. MFI values measured as geometric mean of MHC II antibody (L243) reactive positive cells are shown in bar diagram. The data is from whole blood staining performed on six animals. The data shown include results from six animals (*n* = 6). *p* < 0.0001(****). One‐way ANOVA used to obtain *p* values (GraphPad Prism software v 9.0.0). ANOVA, analysis of variance; MHC, major histocompatibility complex.

To monitor the potential expression of MHC II in SQM immune cells, we first investigated staining of WB with human MHC II antibody. We used L243 a clone of human MHC II antibody,^47^ throughout the study except where indicated. This clone is routinely used in nonhuman primates for MHC II expression^38^, ^48^ and its cross reactivity with NHP immune cells is established.^49^, ^50^ Freshly collected WB (100 µL) was assessed by flow cytometry. The WB was stained with conjugated antibodies for T lymphocytes (CD3), B cells (CD20) monocytes (CD14) along with conjugated MHC II antibody. The details of clones used for each antibody are given in the materials and methods section. Lymphocytes and monocytes gated on scatter plots (FSC/SSC) were used to identify CD3^+^ T lymphocytes, CD20^+^ B cells, and CD14^+^ monocytes, and MHC II class antibody reactive populations were identified as double positive for each population of cells (CD3^+^MHCII^+^, CD20^+^MHCII^+^ and CD14^+^MHCII^+^ cells) cells from quadrant plots. As shown in Figure 1, B cells (CD20^+^) and monocytes (CD14^+^) from SQM peripheral blood showed strong reactivity with MHC class II antibody as tested. Interestingly, CD3^+^ T lymphocytes also showed positive reactivity and >50% of T lymphocytes were positive for expression of MHC II. We measured mean fluorescence intensity (MFI) (as geometric mean) for fluorochrome of MHC II antibody‐stained positive T lymphocytes, B cells and monocytes, from the same test to check density of MHC II molecules expressed. MFI values were higher for B cells showing more MHC II molecules. There is no significant difference in MFI shown by monocytes and T lymphocytes (Figure 2).

### 3.2 Binding of antibody (clone L243) is specific to MHC II

**Figure 3:**
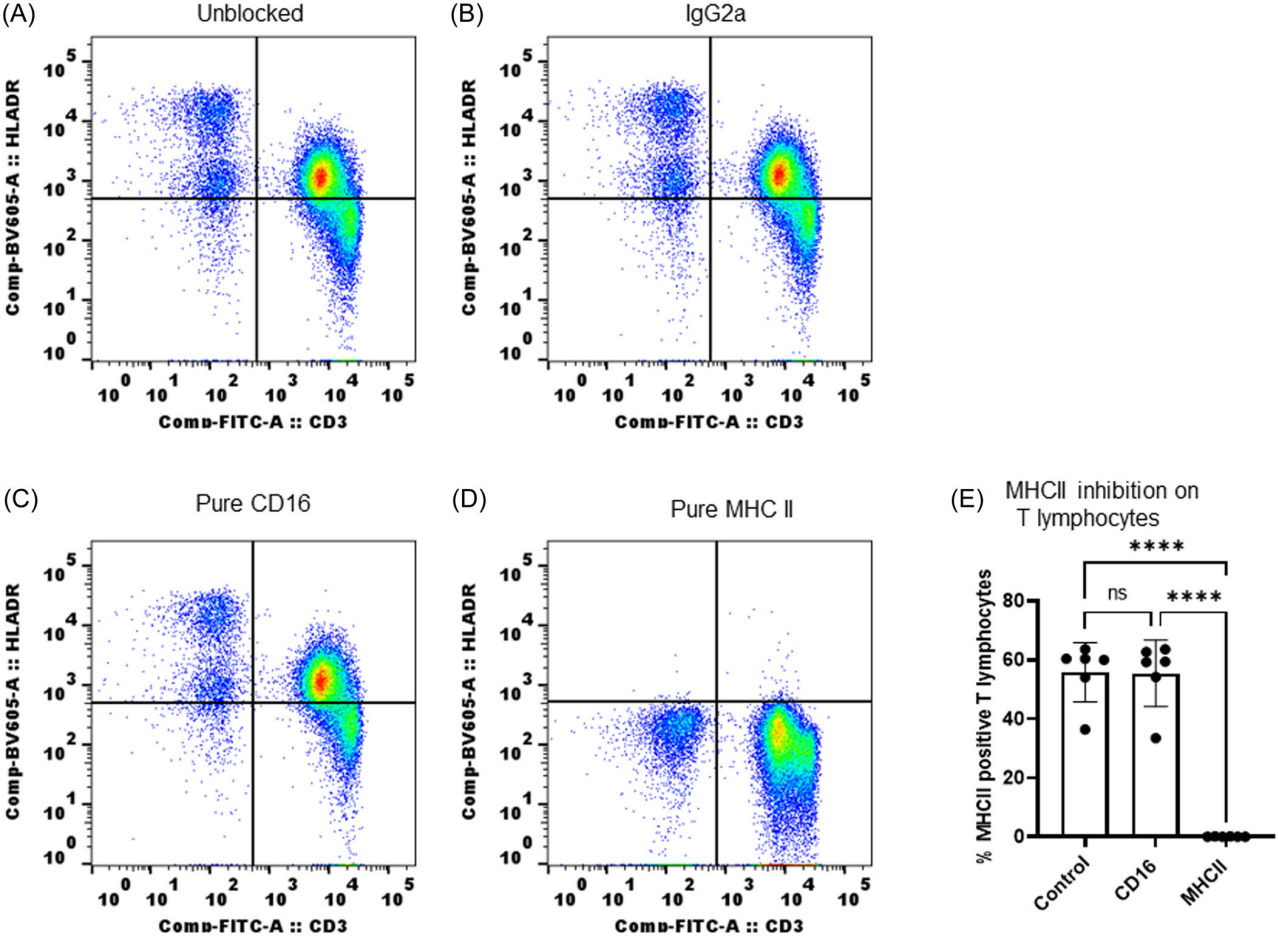
FACS analysis of L243 antibody binding specificity with T lymphocytes: (A) Control; (B) preblocked with isotype IgG2a; (C) preblocked with purified CD16 antibody; (D) inhibition of L243 binding to T lymphocytes by preblocking with purified L243; (E) MHC II inhibition on T lymphocytes shown as bar‐diagram. The data shown include results from six animals (*n* = 6). *p* < .0001(****). One‐way ANOVA used to obtain *p* values (GraphPad Prism software v 9.0.0). ANOVA, analysis of variance; MHC, major histocompatibility complex.

To rule out whether the observed strong reactivity with T lymphocytes was due to nonspecific binding of antibody, we evaluated antibody reactivity in WB samples preincubated with matched isotype (mouse IgG2a), CD16 antibody (clone 3G8), and purified MHC II antibody. WB was incubated with purified antibodies (as listed) for 30 min at RT before stain with fluorochrome conjugated CD3 and MHC II antibodies. Antibody (MHC II) reactivity with T lymphocytes completely disappeared in blood samples that were preincubated with purified antibody (Figure 3A–E). Prior incubation with matching isotype antibody and CD16 antibody was similar as control (WB alone).

### 3.3 SQM T lymphocytes showed reactivity with alternate clone of MHC II antibody LB3.1 and clone L243 binding is blocked by preincubation of WB with similar clone LB3.1

**Figure 4:**
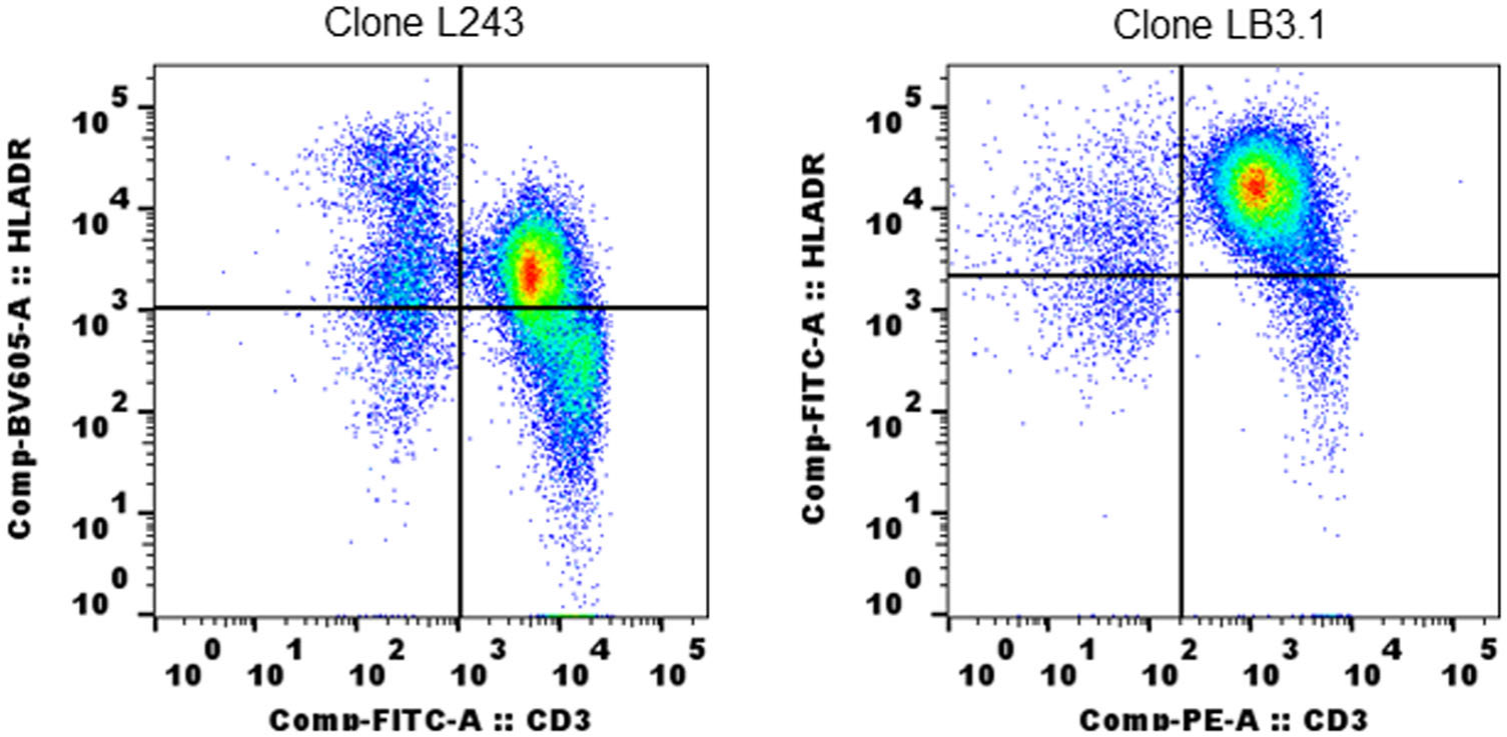
Reactivity of T lymphocytes is similar with two different clones of human MHC II antibodies: SQM whole blood tested with LB3.1 (gifted by Prof Lawrence J Stern) and L243. Both clones were used at the same concentration to stain 100 µL of whole blood and followed same staining protocol described in Section 2. MHC, major histocompatibility complex; SQM, squirrel monkey.

**Figure 5:**
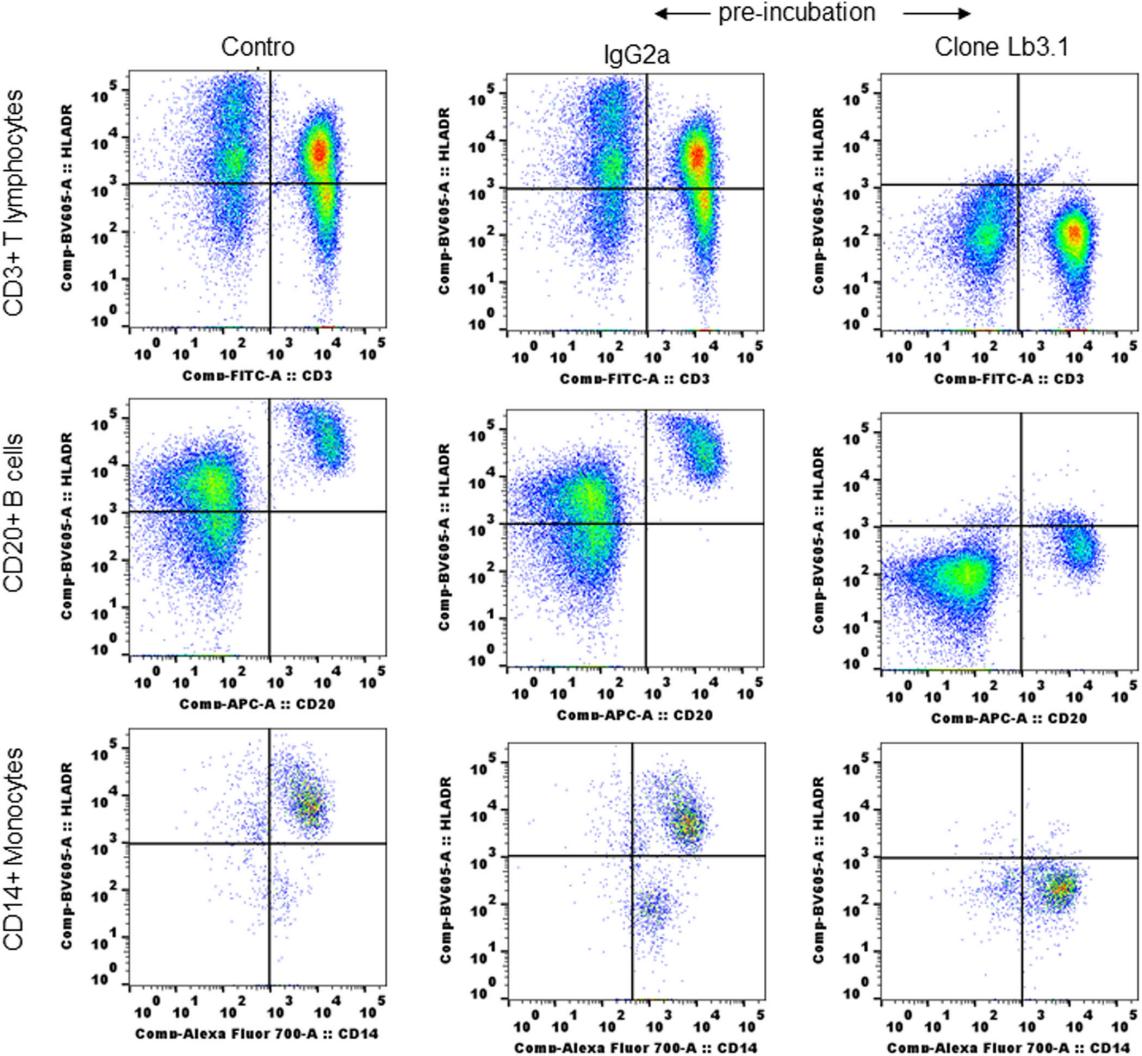
Preincubation of whole blood with clone LB3.1 inhibited reactivity of clone L243: 100 µL SQM whole blood is incubated with 5.0 µg of purified clone LB3.1 for 30 min at RT. After incubation whole blood is stained with conjugated CD3, CD20, CD14, and MHC II (clone L243) antibodies to test reactivity of L243 with different cell populations. The staining protocol, flow cytometry and analysis were described in Section 2. MHC, major histocompatibility complex; RT, room temperature; SQM, squirrel monkey.

We next tested T lymphocyte reactivity with an alternate, but similar clone of MHC II antibody to confirm results from clone L243. The alternate clone LB3.1 also showed binding reactivity with T lymphocytes as seen with L243 (Figure 4). To find whether both clones react using the same epitopes, we preblocked with purified LB3.1 and monitored reactivity with L243 (Figure 5). WB alone incubated with matched isotype (IgG2a) was used as a control. Preincubation of LB3.1 completely blocked L243 reactivity in T lymphocytes as in B cells and monocytes (Figure 5).

### 3.4 MHC class II expression (MHC II antibody reactivity) on T lymphocytes in PBMC from SQM

**Figure 6:**
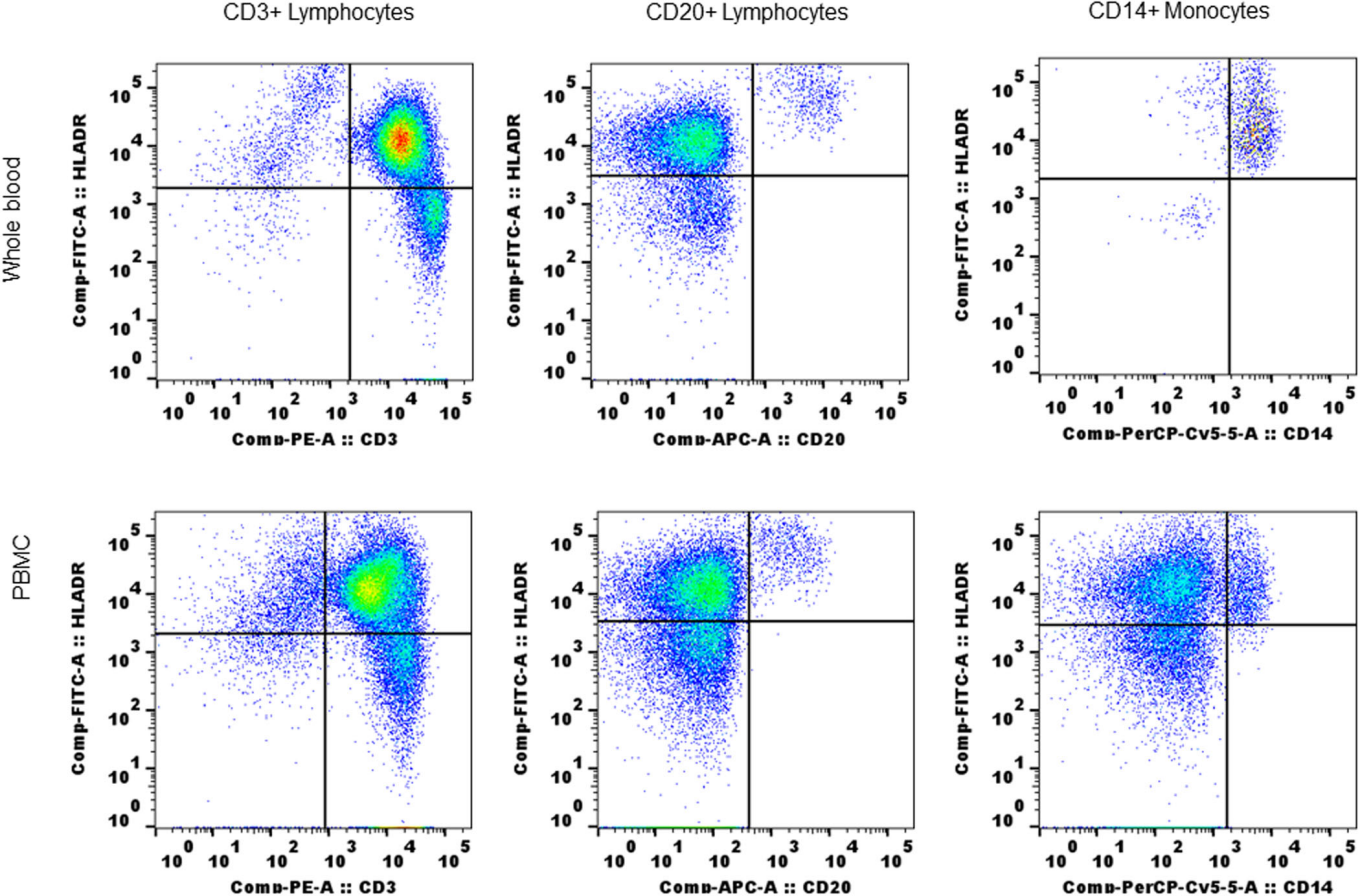
Freshly isolated PBMC retained MHC II antibody reactivity: PBMC were prepared by Ficoll gradient method (see Section 2) and used immediately for staining with cocktail of conjugated CD3, CD20, and CD14 antibodies along with MHC II antibody. Whole blood from same animal which used for PBMC preparation was also tested simultaneously. The details of staining protocol and flow cytometry were as described in Section 2). Whole blood: upper panel; PBMC: lower panel. MHC, major histocompatibility complex; PBMC, peripheral blood mononuclear cell.

Freshly isolated PBMC from SQM blood were evaluated, as done with WB, for reactivity with human MHC II antibody (clone L243). Results from a four color (4C) phenotype flow analysis were shown in Figure 6. The data clearly showed that antibody reactivity with T lymphocytes is not a WB phenomenon, CD3^+^ T cells along with B cells and monocytes showed positive responses in freshly prepared PBMC (in vitro) showing unstimulated T cells express MHC II molecules in SQM.

### 3.5 MHC II expression on T lymphocytes in NWM not seen in OWM

**Figure 7:**
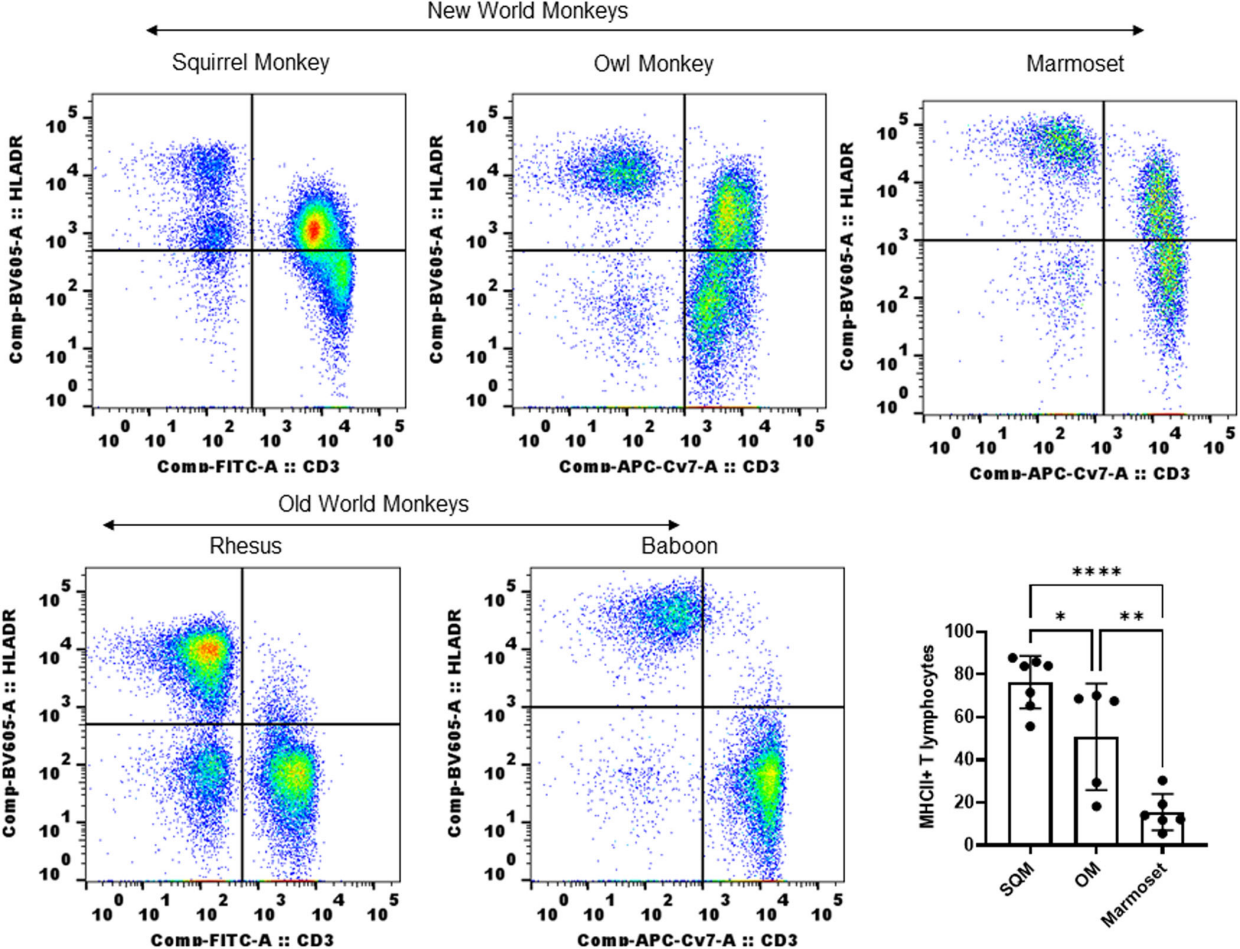
New world monkeys express MHC II antibody reactive molecules on whole blood T lymphocytes: Whole blood from owl monkey, marmoset, rhesus, and baboon was also tested along with squirrel monkey for reactivity with MHC class II antibody (L243). The staining protocol and flow cytometry details were given in Section 2. New world monkeys: upper panel; Old world monkeys: lower panel; The data shown include results from SQM (*n* = 7), OM (*n* = 5), and marmoset (*n* = 6) animals. *p* < 0.0001(****), 0.0060 (**), and 0.0369 (*). One‐way ANOVA used to obtain *p* values (GraphPad Prism software v 9.0.0). ANOVA, analysis of variance; MHC, major histocompatibility complex; OM, owl monkey; SQM, squirrel monkey.

SQM belong within the group of NWM. To verify whether expression of MHC II on T lymphocytes is a common phenotype in NWM, we tested for MHC II expression in owl and marmoset monkeys as other species of NWM along with rhesus macaques and olive baboon as representative species of OWM. The results shown in Figure 7 clearly demonstrate that owl and marmoset monkeys show strongly positive reactivity with T lymphocytes just as seen with SQM. Both rhesus and baboons showed reactivity only with B cells and monocytes, with no traces of reactivity with T lymphocytes (Figure 7). Among the NWM, SQM has more MHC II^+^ T lymphocytes followed by OM and marmoset as seen by antibody reactivity.

## 4 DISCUSSION

In the present study, we looked at the reactivity of human MHC II antibody with immune cells from SQM, OM, and marmoset monkeys to monitor MHC II expression. Our results from both WB and PBMC (SQM) clearly demonstrated strong reactivity of MHC II antibody with T lymphocytes showing expression of MHC II molecules on T lymphocytes in NWM species. We used two standard monoclonal antibodies (clones L243 and LB3.1) and tested their reactivity or binding to immune cells primarily in SQM. As reported in other nonhuman primates, CD20^+^ B cells and CD14^+^ monocytes showed positive reactivity with both clones of MHC II antibody. Interestingly, in SQM T lymphocytes also showed strong reactivity with both clones. The reactivity showed uniformly on a high percentage of T lymphocytes, though MFI is lower than B cells, but comparable to monocytes. When considering MFI (measured as geometric mean of fluorescence in positive cells) as the density of reactive molecules, T lymphocytes in SQM express a low density of MHC II molecules like monocytes, the precursors of DCs which upregulate MHC II molecules expression when activated and differentiated.

To rule out nonspecific reactivity and confirm reactivity is indeed with MHC II molecules, we ran preblocking experiments with purified matching isotype controls using FcgRIIIa (CD16) and MHC II antibodies before staining with conjugated MHC II antibody to check MHC II expression. CD16 is a transmembrane form of the low‐affinity Fc receptor for IgG (CD16, FcgRIIIa) and its positive expression on T lymphocytes is used as a marker for natural killer T (NKT) cells in NWM.^9^, ^11^, ^39^, ^40^, ^51^ We reasoned interference of surface expressed CD16 for potential nonspecific binding of MHC II antibody as both CD16 and HLADR are expressed on WB lymphocytes, hence we included purified CD16 antibody (clone 3G8) in preblocking assays. The experiments clearly showed that binding of both antibodies (CD16 and MHC II) has different specificity and has no interference of one over the other, as we noticed similar T lymphocytes reactivity of MHC II antibody as control in CD16 preblocked samples. The complete inhibition of conjugated antibody with T lymphocytes preblocked with either purified antibody clone (L243 and LB3.1) shows that reactivity is specific to MHC II molecules expressed on T lymphocytes. Further confirmation comes from inhibition of antibody binding in B cells and monocytes, the traditional MHC II positive immune cells. The preblocking experiments also proved both clones bind well and might recognize the same epitopes on MHC II for binding.

Though WB was routinely used as a sample type in our studies, to confirm that expression of MHC II is not limited to WB samples, we tested using freshly isolated PBMC. T lymphocytes along with B cells and monocytes showed MHC II antibody reactivity in SQM PBMC, without any stimulation. Contamin et al.^38^ showed HLADR expression on lymphocytes (CD4) from SQM PBMC stimulated with PHA‐L. Our results are the first to show constitutive expression of MHC II molecules on T lymphocytes in SQM and other New World monkeys (OM and marmoset), without any stimulation or infection. It is interesting to verify whether T lymphocytes in these monkeys are naturally activated due to presence of indigenous viruses carried by these monkeys. Also based on our results we will investigate further whether in vitro activation (IFNγ or other cytokines) will enhance MHC II expression on T lymphocytes from SQM.

In general, MHC II molecules are loaded with processed peptides and are stably expressed on APC. Using monoclonal antibodies, MHC II molecules associated either with invariant chain, CLIP or no peptide (empty MHC) were identified on professional and nonprofessional APC or mutants.^9^, ^11^, ^51^ These variants of stable MHC II molecules expressed on professional APCs have capacity for peptide loading (either by exchange or uptake) and presentation.^52^, ^53^ Cytokine stimulated T lymphocytes, long term viral infected T lymphocytes, malignant T lymphocytes, and resting T lymphocytes expressing MHC II molecules function as APCs with expression of costimulatory molecules such as CD80.^54^ However antigen presentation mechanism by T lymphocytes is not clearly defined or well understood.^55^ Our work is limited at this time to further phenotype MHCII^+^ T lymphocytes and functional studies, due to limited availability of cross‐reactive reagents (CD45, CD45RA, CD8 other marker antibodies), yet this definitive report on MHC II expression on T lymphocytes in NWM requires further analyzes including detailed phenotype of MHCII^+^ T lymphocytes, effect of aging, understanding the nature of surface expressed MHC II molecules and whether participate in immune functions. Marmoset monkeys are unique in their expression of MHC II on neutrophils which is not shared by other nonhuman primates.^56^ These studies, combined with our results, show that NWM are unique in expression of MHC II molecules on different immune cells. We need to perform added studies to further characterize the functional and evolutionary significance of these findings to better understand NWM as models in biomedical research.

## AUTHOR CONTRIBUTIONS

**Sriram Chitta**: Conceptualization, resouces, methodology, investigation, data analysis, writing original draft, review and editing. **Bharti P. Nehete**: resources, investigation, writing‐review and editing. **Ashley B. Delise**: investigation, writing‐review and editing. **Joe H. Simmons**: resources, funding, writing‐review and editing. **Pramod N. Nehete**: Conceptualization, data analysis, investigation, methodology; resources, writing‐review and editing.

## CONFLICT OF INTEREST STATEMENT

The authors declare no conflicts of interest.

## ETHICS STATEMENT

This research was conducted at the AAALAC‐accredited Michale E Keeling Center for Comparative Medicine and Research (Keeling Center) at the University of Texas MD Anderson Cancer Center (Bastrop, TX). All blood samples were collected as part of routine veterinary medical examination and according to the provisions of the Animal Welfare Act, PHS Animal Welfare Policy, and the principles of the NIH Guide for the Care and Use of Laboratory Animals.^57^ All procedures were approved by the IACUC (ACUF 00000451‐RN04) at the University of Texas MD Anderson Cancer Center. The Keeling Center has been fully accredited continuously since 1979 by AAALAC.

## Data Availability

The data presented in the study are available from the corresponding author upon request.

## References

[1] Cresswell P . Assembly, transport, and function of MHC class II molecules. Annu Rev Immunol. 1994;12:259‐291. 8011283 10.1146/annurev.iy.12.040194.001355

[2] Watts C . The exogenous pathway for antigen presentation on major histocompatibility complex class II and CD1 molecules. Nature Immunol. 2004;5(7):685‐692. 15224094 10.1038/ni1088

[3] Chesnut RW , Grey HM . Antigen presenting cells and mechanisms of antigen presentation. Crit Rev Immunol. 1985;5(3):263‐316. 3884274

[4] Sallusto F , Lanzavecchia A . Efficient presentation of soluble antigen by cultured human dendritic cells is maintained by granulocyte/macrophage colony‐stimulating factor plus interleukin 4 and downregulated by tumor necrosis factor alpha. J Exp Med. 1994;179(4):1109‐1118. 8145033 10.1084/jem.179.4.1109 PMC2191432

[5] Randolph GJ , Beaulieu S , Lebecque S , Steinman RM , Muller WA . Differentiation of monocytes into dendritic cells in a model of transendothelial trafficking. Science. 1998;282(5388):480‐483. 9774276 10.1126/science.282.5388.480

[6] Sugimoto C , Hasegawa A , Saito Y , et al. Differentiation kinetics of blood monocytes and dendritic cells in macaques: insights to understanding human myeloid cell development. J Immunol. 2015;195(4):1774‐1781. 26179903 10.4049/jimmunol.1500522 PMC4530075

[7] O'Doherty U , Ignatius R , Bhardwaj N , Pope M . Generation of monocyte‐derived dendritic cells from precursors in rhesus macaque blood. J Immunol Methods. 1997;207(2):185‐194. 9368645 10.1016/s0022-1759(97)00119-1

[8] Nehete PN , Chitta S , Hossain MM , et al. Protection against chronic infection and AIDS by an HIV envelope peptide‐cocktail vaccine in a pathogenic SHIV‐rhesus model. Vaccine. 2001;20(5‐6):813‐825. 11738745 10.1016/s0264-410x(01)00408-x

[9] Eastman S , Deftos M , DeRoos PC , et al. A study of complexes of class II invariant chain peptide: major histocompatibility complex class II molecules using a new complex‐specific monoclonal antibody. Eur J Immunol. 1996;26(2):385‐393. 8617308 10.1002/eji.1830260218

[10] Avva RR , Cresswell P . In vivo and in vitro formation and dissociation of HLA‐DR complexes with invariant chain‐derived peptides. Immunity. 1994;1(9):763‐774. 7895165 10.1016/s1074-7613(94)80018-9

[11] Santambrogio L , Sato AK , Fischer FR , Dorf ME , Stern LJ . Abundant empty class II MHC molecules on the surface of immature dendritic cells. Proc Natl Acad Sci USA. 1999;96(26):15050‐15055. 10611336 10.1073/pnas.96.26.15050 PMC24771

[12] Carven GJ , Chitta S , Hilgert I , et al. Monoclonal antibodies specific for the empty conformation of HLA‐DR1 reveal aspects of the conformational change associated with peptide binding. J Biol Chem. 2004;279(16):16561‐16570. 14757758 10.1074/jbc.M314315200

[13] Chitta S , Santambrogio L , Stern LJ . GMCSF in the absence of other cytokines sustains human dendritic cell precursors with T cell regulatory activity and capacity to differentiate into functional dendritic cells. Immunol Lett. 2008;116(1):41‐54. 18166231 10.1016/j.imlet.2007.11.013

[14] Holling TM , Schooten E , van Den Elsen PJ . Function and regulation of MHC class II molecules in T‐lymphocytes: of mice and men. Hum Immunol. 2004;65(4):282‐290. 15120183 10.1016/j.humimm.2004.01.005

[15] Ko HS , Fu SM , Winchester RJ , Yu DT , Kunkel HG . Ia determinants on stimulated human T lymphocytes. occurrence on mitogen‐ and antigen‐activated T cells. J Exp Med. 1979;150(2):246‐255. 88499 10.1084/jem.150.2.246 PMC2185627

[16] Ho HN , Hultin LE , Mitsuyasu RT , et al. Circulating HIV‐specific CD8+ cytotoxic T cells express CD38 and HLA‐DR antigens. J Immunol. 1993;150(7):3070‐3079. 8454874

[17] Tippalagama R , Singhania A , Dubelko P , et al. HLA‐DR marks recently divided antigen‐specific effector CD4 T cells in active tuberculosis patients. J Immunol. 2021;207(2):523‐533. 34193602 10.4049/jimmunol.2100011 PMC8516689

[18] Baecher‐Allan C , Wolf E , Hafler DA . MHC class II expression identifies functionally distinct human regulatory T cells. J Immunol. 2006;176(8):4622‐4631. 16585553 10.4049/jimmunol.176.8.4622

[19] Holling TM , Schooten E , Langerak AW , van den Elsen PJ . Regulation of MHC class II expression in human T‐cell malignancies. Blood. 2004;103(4):1438‐1444. 14563641 10.1182/blood-2003-05-1491

[20] Reizis B , Schramm C , Cohen IR , Mor F . Expression of major histocompatibility complex class II molecules in rat T cells. Eur J Immunol. 1994;24(11):2796‐2802. 7525305 10.1002/eji.1830241133

[21] Doveren RFC , van der Linden CJ , Spronken EEM , Groenewegen G , Buurman WA . Canine MHC‐class II antigens on B and T lymphocytes. Tissue Antigens. 1986;27(2):87‐98. 3518148 10.1111/j.1399-0039.1986.tb01503.x

[22] Bendali‐Ahcène S , Cadore JL , Fontaine M , Monier JC . Anti‐alpha chain monoclonal antibodies of equine MHC class‐II antigens: applications to equine infectious anaemia. Res Vet Sci. 1997;62(2):99‐104. 9243705 10.1016/s0034-5288(97)90128-4

[23] Lunn DP , Holmes MA , Duffus WPH . Equine T‐lymphocyte MHC II expression: variation with age and subset. Vet Immunol Immunopathol. 1993;35(3‐4):225‐238. 8094263 10.1016/0165-2427(93)90036-4

[24] Saalmüller A , Weiland F , Reddehase MJ . Resting porcine T lymphocytes expressing class II major histocompatibility antigen. Immunobiology. 1991;183(1‐2):102‐114. 1834544 10.1016/S0171-2985(11)80190-7

[25] Isaacson JA , Flaming KP , Roth JA . Increased MHC class II and CD25 expression on lymphocytes in the absence of persistent lymphocytosis in cattle experimentally infected with bovine leukemia virus. Vet Immunol Immunopathol. 1998;64(3):235‐248. 9730219 10.1016/s0165-2427(98)00139-1

[26] Chang CH , Hong SC , Hughes CCW , Janeway Jr. CA , Flavell RA . CIITA activates the expression of MHC class II genes in mouse T cells. Int Immunol. 1995;7(9):1515‐1518. 7495759 10.1093/intimm/7.9.1515

[27] Slierendregt BL , Bontrop RE . Current knowledge on the major histocompatibility complex class II region in non‐human primates. Int J Immunogenet. 1994;21(5):391‐402. 10.1111/j.1744-313x.1994.tb00212.x 9098449

[28] Charron DJ , McDevitt HO . Analysis of HLA‐D region‐associated molecules with monoclonal antibody. Proc Natl Acad Sci USA. 1979;76(12):6567‐6571. 392522 10.1073/pnas.76.12.6567 PMC411907

[29] Charron DJ , McDevitt HO . Characterization of HLA‐D‐region antigens by two‐dimensional gel electrophoresis. Molecular‐genotyping. J Exp Med. 1980;152(2 Pt 2):18s‐36s. 6931876

[30] Andrews DW , Bono MR , Kaufman JF , Knudsen P , Strominger JL . Use of monoclonal antibody immunoaffinity columns to purify subsets of human HLA‐DR antigens. Methods Enzymol. 1984;108:600‐606. 6597333 10.1016/s0076-6879(84)08120-9

[31] Lekutis C , Letvin NL . Biochemical and molecular characterization of rhesus monkey major histocompatibility complex class II DR. Hum Immunol. 1995;43(1):72‐80. 7558932 10.1016/0198-8859(94)00155-j

[32] Manning CH , Heise ER . Biochemical analysis of class I and class II MHC antigens in cynomolgus macaques by one‐dimensional isoelectric focusing. Tissue Antigens. 1991;37(2):56‐65. 1905425 10.1111/j.1399-0039.1991.tb01846.x

[33] Slierendregt BL , Otting N , Jonker M , Bontrop RE . Gel electrophoretic analysis of rhesus macaque major histocompatibility complex class II DR molecules. Hum Immunol. 1994;40(1):33‐40. 8045791 10.1016/0198-8859(94)90019-1

[34] O'hUigin C . Nonhuman primate MHC class II sequences: a compilation. Molecular Biology and Evolution of Blood Group and MHC Antigens in. Primates. Springer; 1997:507‐551.

[35] Bontrop R . MHC class II genes of nonhuman primates: the DR loci. Molecular biology and evolution of blood group and MHC antigens in primates. Springer; 1997:358‐371.

[36] Klein J , Satta Y , Gongora R . Evolution of length variation in the primate MHC DR subregion. Molecular biology and evolution of blood group and MHC antigens in primates. Springer; 1997:372‐385.

[37] Ozwara H , Niphuis H , Buijs L , et al. Flow cytometric analysis on reactivity of human T lymphocyte‐specific and cytokine‐receptor‐specific antibodies with peripheral blood mononuclear cells of chimpanzee (*Pan troglodytes*), rhesus macaque (*Macaca mulatta*), and squirrel monkey (*Saimiri sciureus*). J Med Primatol. 1997;26(3):164‐171. 9379483 10.1111/j.1600-0684.1997.tb00048.x

[38] Contamin H , Loizon S , Bourreau E , et al. Flow cytometry identification and characterization of mononuclear cell subsets in the neotropical primate *Saimiri sciureus* (squirrel monkey). J Immunol Methods. 2005;297(1‐2):61‐71. 15777931 10.1016/j.jim.2004.11.019

[39] Nehete PN , Hanley PW , Nehete BP , et al. Phenotypic and functional characterization of lymphocytes from different age groups of bolivian squirrel monkeys (*Saimiri boliviensis* boliviensis). PLoS One. 2013;8(11):e79836. 24282512 10.1371/journal.pone.0079836 PMC3839916

[40] Nehete PN , Nehete BP , Chitta S , Williams LE , Abee CR . Phenotypic and functional characterization of peripheral blood lymphocytes from various age‐ and sex‐specific groups of owl monkeys (*Aotus nancymaae*). Comp Med. 2017;67(1):67‐78. 28222841 PMC5310627

[41] Nehete PN , Shelton KA , Nehete BP , et al. Effects of transportation, relocation, and acclimation on phenotypes and functional characteristics of peripheral blood lymphocytes in rhesus monkeys (*Macaca mulatta*). PLoS One. 2017;12(12):e0188694. 29261698 10.1371/journal.pone.0188694 PMC5736198

[42] de Oliveira EHC , Neusser M , Müller S . Chromosome evolution in new world monkeys (Platyrrhini). Cytogenet Genome Res. 2012;137(2‐4):259‐272. 22699158 10.1159/000339296

[43] Shelton KA , Nehete BP , Chitta S , et al. Effects of transportation and relocation on immunologic measures in cynomolgus macaques (*Macaca fascicularis*). J Am Assoc Lab Anim Sci. 2019;58(6):774‐782. 31604484 10.30802/AALAS-JAALAS-19-000007 PMC6926399

[44] Magden ER , Nehete BP , Chitta S , et al. Comparative analysis of cellular immune responses in conventional and SPF olive baboons (*Papio anubis*). Comp Med. 2020;70(2):160‐169. 32014083 10.30802/AALAS-CM-19-000035 PMC7137550

[45] Bontrop RE , Elferink DG , Otting N , Jonker M , de Vries RR . Major histocompatibility complex class II‐restricted antigen presentation across a species barrier: conservation of restriction determinants in evolution. J Exp Med. 1990;172(1):53‐59. 1694228 10.1084/jem.172.1.53 PMC2188146

[46] Geluk A , Elferink DG , Slierendregt BL , et al. Evolutionary conservation of major histocompatibility complex‐DR/peptide/T cell interactions in primates. J Exp Med. 1993;177(4):979‐987. 8459225 10.1084/jem.177.4.979 PMC2190985

[47] Lampson LA , Levy R . Two populations of Ia‐like molecules on a human B cell line. J Immunol. 1980;125(1):293‐299. 6966655

[48] Kap YS , van Meurs M , van Driel N , et al. A monoclonal antibody selection for immunohistochemical examination of lymphoid tissues from non‐human primates. J Histochem Cytochem. 2009;57(12):1159‐1167. 19729671 10.1369/jhc.2009.954123 PMC2778089

[49] Neumann B , Shi T , Gan LL , et al. Comprehensive panel of cross‐reacting monoclonal antibodies for analysis of different immune cells and their distribution in the common marmoset (*Callithrix jacchus*). J Med Primatol. 2016;45(3):139‐146. 27221549 10.1111/jmp.12216

[50] Bjornson‐Hooper ZB , Fragiadakis GK , Spitzer MH , et al. Cell type‐specific monoclonal antibody cross‐reactivity screening in non‐human primates and development of comparative immunophenotyping panels for CyTOF. Biorxiv. 2019:577759. 10.1101/577759

[51] Veenstra H , Ferris WF , Bouic PJD . Major histocompatibility complex class II invariant chain expression in non‐antigen‐presenting cells. Immunology. 2001;103(2):218‐225. 11412309 10.1046/j.1365-2567.2001.01230.x PMC1783233

[52] Venkatraman P , Nguyen TT , Sainlos M , et al. Fluorogenic probes for monitoring peptide binding to class II MHC proteins in living cells. Nat Chem Biol. 2007;3(4):222‐228. 17351628 10.1038/nchembio868 PMC3444530

[53] Santambrogio L , Sato AK , Carven GJ , Belyanskaya SL , Strominger JL , Stern LJ . Extracellular antigen processing and presentation by immature dendritic cells. Proc Natl Acad Sci USA. 1999;96(26):15056‐15061. 10611337 10.1073/pnas.96.26.15056 PMC24772

[54] Saalmüller A , Maurer S . Major histocompatibility antigen class II expressing resting porcine T lymphocytes are potent antigen‐presenting cells in mixed leukocyte culture. Immunobiology. 1994;190(1‐2):23‐34. 8082885 10.1016/S0171-2985(11)80281-0

[55] Pichler WJ , Wyss‐Coray T . T cells as antigen‐presenting cells. Immunol Today. 1994;15(7):312‐315. 7522009 10.1016/0167-5699(94)90078-7

[56] Ngugi S , Laws T , Simpson AJ , Nelson M . The innate immune response in the marmoset during the acute pneumonic disease caused by burkholderia pseudomallei. Infect Immun. 2022;90(3):e0055021. 35041487 10.1128/iai.00550-21 PMC8929355

[57] Council NR . Guide for the Care and Use of Laboratory Animals. Eighth Edition. The National Academies Press; 2011:246. 21595115

